# Correspondence

**Published:** 1874-08

**Authors:** Wm. T. Beall

**Affiliations:** Edwards, Miss.


EDWARDS, MISS., April 27, 1874.

*Messrs. Editors:*

I have recently become a subscriber to the RECORD, and though unaccustomed to writing for journals, am induced by an article in the February number headed "Gelseminum," by Dr. Hoge, of Arkansas, to venture a few remarks upon the treatment of the malarious fevers of our country, particularly that type termed "remittent." An experience of twenty years in the swamps of Mississippi has satisfied me that the great secret of early success in arresting these fevers, is the proper *time* for the use of sulph. quinia. This time I regard as at one o'clock at night, and so well assured am I of this fact, that I universally prescribe quinine at that hour, to be repeated every two hours until enough is given, regardless of the condition of my patient. I say regardless, because observation has satisfied me that the remission is more complete at that hour, or beeween 12 and 2 o'clock at night, than at any other time in the twenty-four hours. Nor do I leave it to be given when mercurials, purgatives or other remedies have acted; for in many cases the physician has not time to secure the action of such remedies in time to arrest the next paroxysm. If the stomach is too irritable to take it, I give it by enema; but I have found that quinine will often quiet the nausea and vomiting of bilious fever when nothing else will.

Then, to conclude, I am satisfied if we would treat these fevers more at night, and leave the prescription, quinine, at one o'clock, fever or no fever, and not leave it to the judgement of nurses to detect the remission, we would save ourselves a great deal of trouble and our patients a vast deal of suffering. Respectfully,

WM. T. BEALL.

